# Report of Case

**Published:** 1893-07

**Authors:** C. J. Carter

**Affiliations:** Lake Park, Ga.


### REPORT OF CASE.

BY C. J. CARTER, M. D., LAKE PARK, GA.

On February 23d I was called to see a patient eight miles in the country. Found on arrival a young man, age 25, who on February 15th was taken with pain in his stomach and had to quit his work. In the course of a few hours had a chill, followed by two or three lighter ones. On the 17th and 18th he had high fever and a physician was sent for and pronounced the case, one of bilious fever. Bowels constipated and pain in side continuous. On the 21st he rode nine miles in a buggy to see his physician, who gave him a bottle of bitters and a laxative.

The above is a short history of the case as given me by patient and nurse on the 24th, the time at which I first saw patient.

Finding that I had been called to see the patient of another physician, I hesitated about making an examination, but owing to distance of attending physician, I examined the patient. He showed no marked evidence of great suffering. Tongue lightly coated, large and white with indentation of teeth on sides.

Temperature 101°, had rested fairly well night before. Appetite poor. Upon examination, I found right side of abdomen rather more full than left and on palpation discovered a tumor about the region of the vermiform appendix. The entire right iliac region was quite painful and sensitive to the touch. I prescribed sulphate of magnesia, to be given until the bowels were moved, and directed morphine to be given during night if pain became very severe. Directed them to send for the attending physician and tell him to come prepared to operate. I promising to meet him early next morning.

The following morning, early, I met the attending physician. We found that the patient had spent a restless night, and there seemed now to be a general peritonitis. The tumor which I had discovered in the right iliac region, had disappeared. Upon introducing an aspirating needle at the point where the tumor was most prominent the day before, pus escaped freely and we determined to operate at once.

Upon opening the obdominal cavity, there flowed out probably three pints of pus. The patient showing signs of giving way under the anæsthetic, we put in a large rubber drainage tube, dressed the wound and got patient in bed about 11 o'clock a. m. This was on the 24th. Patient died at 5 a. m. on 25th. No autopsy.

